# ID 119 - Teste de Detecção de HLA-B27 para Indivíduos com Suspeita de Espondiloartrite Axial no SUS: revisão sistemática, custo-efetividade e impacto orçamentário

**DOI:** 10.5327/2237-9622.2025.v34s1.119

**Published:** 2025-11-25

**Authors:** Vinicius Lins Ferreira, Layssa Andrade Oliveira, Haliton Alves Oliveira, Rosa Camila Lucchetta

**Keywords:** espondiloartrite axial, protocolos clínicos, diagnóstico clínico, antígeno HLA-B27

## Abstract

**Introdução:**

A espondiloartrite axial (EpA) é uma doença inflamatória crônica, de origem autoimune, caracterizada por dor intensa, enrijecimento das articulações com inflamação nas inserções dos tendões, limitação funcional progressiva, provocando danos estruturais irreversíveis das articulações sacroilíacas e espinhais, que cursam com alterações radiográficas e formação óssea excessiva. O antígeno HLA-B27 está fortemente correlacionado com o aparecimento da doença e um teste positivo para esse marcador é encontrado na maioria dos casos. No Brasil, diversos estudos sugerem que os portadores do antígeno HLA-B27 representam em torno de 60%-70% dos pacientes. No SUS, a avaliação é feita considerando os critérios ASAS, de Nova Iorque, e diagnóstico por radiologista, principalmente. O objetivo deste estudo foi de avaliar a acurácia, a custo-efetividade e o impacto orçamentário do procedimento na perspectiva do SUS.

**Método:**

Uma revisão sistemática foi conduzida para avaliar as evidências do teste, com buscas conduzidas no PubMed, Embase, LILACS e Cochrane Library. A avaliação do risco de viés foi realizada utilizando a ferramenta QUADAS-2 e a qualidade das evidências foi graduada conforme o sistema GRADE. Além disso, uma análise de custo-efetividade (ACE; modelo de árvore de decisão acoplada a um modelo de Markov; horizonte temporal lifetime; desfecho de efetividade: ano de vida ajustado à qualidade – Avaq; taxa de desconto de 5%). A análise de impacto orçamentário (AIO) também foi desenvolvida considerando abordagem da demanda aferida e epidemiológica, e de HLA-B27 variando de 20% a 100%.

**Resultados:**

Nos 29 estudos incluídos, foram comparados o teste HLA-B27 e os critérios de diagnóstico ASAS ou Nova Iorque. A sensibilidade do teste HLA-B27 foi de 68% (IC 95%: 67%-69%) e a especificidade de 88% (IC 95%: 87%-88%), apesar da qualidade da evidência muito baixa. A partir da análise de dois estudos, observou-se que a associação de HLA-B27 e parâmetros clínicos ainda possibilitou o alcance de uma sensibilidade e especificidade comparável ou maior do que a combinação de exame de imagem (ressonância ou radiografia) e parâmetros clínicos, ou apenas os parâmetros clínicos. Em relação à ACE, na comparação com avaliação clínica, o teste HLA-B27 + avaliação clínica foi ligeiramente maior que o limiar de custo-efetividade estabelecido pela Conitec (R$ 43 mil reais por Avaq). Na comparação com avaliação clínica ± exame de imagem, o teste de HLA-B27 + avaliação clínica dominou o comparador. Além disso, observou-se que a incorporação de HLA-B27 no SUS para indicação proposta teria como resultado um incremento de custos de R$ 638 mil no primeiro ano, chegando a R$ 770 mil no quinto ano de análise para uma população elegível variado de 5.748 a 6.937.

**Conclusão:**

Considerando os achados clínicos e econômicos, a adição do teste de HLA-B27 foi considerada benéfica por potencialmente aumentar a acurácia, ser custo-efetiva ou dominante e com impacto orçamentário viável para o SUS. Este estudo subsidiou a recomendação e decisão de incorporação do teste no SUS para indivíduos com suspeita de espondiloartrite axial.

